# Correction: Effect of methotrexate use on the development of type 2 diabetes in rheumatoid arthritis patients: A systematic review and meta-analysis

**DOI:** 10.1371/journal.pone.0243960

**Published:** 2020-12-09

**Authors:** Leena R. Baghdadi

The following information is missing from the Funding statement: This study was funded in part by the Deputyship for Research and Innovation, ‘Ministry of Education’ in Saudi Arabia through the Project no. IFKSURP-160. No additional external funding was received for this study.

The following information is missing from the Acknowledments section: The author extends their appreciation to the Deputyship for Research and Innovation, ‘Ministry of Education’ in Saudi Arabia for funding this research work through the Project no. IFKSURP-160. Special thanks for Dr Emad Mahmoud, an assistant professor, and senior researcher at King Saud University, for the support in evaluating the eligibility of the articles for inclusion in this systematic review and meta-analysis.
