# Supplementary material for: Effect of methotrexate use on the development of type 2 diabetes in rheumatoid arthritis patients: A systematic review and meta-analysis
Source: PLoS One. 2020 Jul 6;15(7):e0235637. doi: 10.1371/journal.pone.0235637 (PMC7337336; doi:10.1371/journal.pone.0235637)
Supplement: S1 Materials — (DOC) [file pone.0235637.s002.doc]

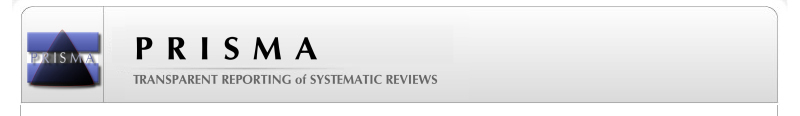
**PRISMA 2009 Flow Diagram**

**Screening**

**Included**

**Eligibility**

**Identification**

Records identified through database searches
(n =4,310)

Additional records identified through handsearching
(n =1)

Records after duplicates removed
(n = 4,170)

Records screened
(n = 4,170)

Records excluded
(n = 4,076)

Full-text articles assessed for eligibility
(n = 94)

Full-text articles excluded,
(n = 78)

- Seventy-five articles excluded as no data available for methotrexate or type 2 diabetes
- One article written in German is excluded due to translation difficulties
- Two articles excluded as no data for rheumatoid arthritis

Studies included in qualitative synthesis
(n = 16)

Studies included in quantitative synthesis (meta-analysis)
(n = 16)
