# Supplementary material for: Effect of methotrexate use on the development of type 2 diabetes in rheumatoid arthritis patients: A systematic review and meta-analysis
Source: PLoS One. 2020 Jul 6;15(7):e0235637. doi: 10.1371/journal.pone.0235637 (PMC7337336; doi:10.1371/journal.pone.0235637)
Supplement: S3 Materials — Data sources. (DOCX) [file pone.0235637.s004.docx]

**Appendix 1.** **Data sources**

**MEDLINE**

(((arthriti* AND rheumatoid) OR Arthritis, Rheumatoid[Mesh]) AND ((Methotrexate[Mesh] OR Methotrexate OR ‎Anti-Rheumatic OR DMARD OR disease modifying antirheumatic drug OR DMARD[mesh] OR Antirheumatic Agents OR Arthritis, Rheumatoid/drug therapy AND Diabetes Mellitus, Type 2[mh] OR (diabetes AND type 2 OR type II))))

**Scopus**

(arthriti* AND rheumatoid)

AND

(diabetes AND (“type 2” OR “type II”))

AND

(methotrexate AND (“anti-rheumatic” OR “dmard”))

AND

(patient* OR person* OR human* OR subjects OR male* OR female*)

**Cochrane Library**

#1 MeSH descriptor: [Arthritis, Rheumatoid] explode all trees

#2 (arthriti* and rheumatoid)

#3 #1 or #2

#4 MeSH descriptor: [Diabetes Mellitus, Type 2] explode all trees

#5 MeSH descriptor: [methotrexate] explode all trees

#6 #3 and #4 and #5
