# Supplementary material for: Effect of methotrexate use on the development of type 2 diabetes in rheumatoid arthritis patients: A systematic review and meta-analysis
Source: PLoS One. 2020 Jul 6;15(7):e0235637. doi: 10.1371/journal.pone.0235637 (PMC7337336; doi:10.1371/journal.pone.0235637)
Supplement: S4 Materials — Checklist for assessing quality of included studies in the meta-analysis. (DOCX) [file pone.0235637.s005.DOCX]

**Appendix 2. Checklist for assessing quality of included studies in the meta-analysis**

| A. | Design-specific control of bias |
| --- | --- |
| 1 | Was a method of randomisation performed? |
|  | 0 = No or not reported |
|  | 1 = yes but allocation not concealed |
|  | 2 = Yes and allocation concealed |
| 2 | Was the data prospectively collected |
|  | 0 = no – case-control or cross-sectional design |
|  | 0.5= no – cohort design |
|  | 1=yes |
| B. | Selection bias |
| 3 | Was the outcome of interest already present at the start of the study? |
|  | 0 = Yes |
|  | 1 = No |
| 4 | Were protocol deviations, losses to follow-up, and drop-out rates acceptable (<20%)? |
|  | 0 = No or not reported |
|  | 0.5 = In part |
|  | 1 = Yes |
| 5 | Were the controls or non-exposed cohort drawn from the same population and in the same way as the exposed cohort? |
|  | 0 = No or no description |
|  | 1 = Drawn from a different source |
|  | 2 = Yes |
| 6 | Were the eligibility criteria clearly specified and uniformly applied to comparison groups |
|  | 0 = No or no description |
|  | 0.5 = In part |
|  | 1 = Yes |
| 7 | Was selection of a comparison group appropriate? |
|  | 0 = No or no description |
|  | 0.5 = In part |
|  | 1 = Yes |
| C. | Confounding |
| 8 | Was any attempt made to balance allocation between groups (excludes randomisation)? |
|  | 0 = No |
|  | 0.5 = in part |
|  | 1 = yes |
| 9 | Were the important prognostic indicators (age, sex, hypertension, BMI, dyslipidaemia, family history of T2D and CVD, physical inactivity, duration of RA, and using medications (folic acid, corticosteroids, traditional and biological DMARDs) of the group/cohorts comparable at baseline and were reported? Were they similar? |
|  | 0 = not reported or 0,1,2 |
|  | 0.5 = 3, 4 |
|  | 1 = 5 or more |
| D. | Information bias |
| 10 | Was there a clear ascertainment of exposure (cohort) or for outcomes or for interventions and were they clearly defined and precisely reported? |
|  | 0 = No description |
|  | 0.5 = Self report for exposure or description in part |
|  | 1 = Yes (e.g. secure record for exposure or structured interview in case of an observational study) |
| 11 | Was timing of outcome assessment in both groups and duration of follow-up comparable and adequate for outcomes to occur? |
|  | 0 = No or not reported |
|  | 0.5 = In part |
|  | 1 = Yes |
| 12 | Were there variations from study protocol that could have affected study measurements? |
|  | 0 = No or not reported |
|  | 0.5 = In part |
|  | 1 = Yes |
| 13 | Were the outcome assessor, care provider, and patients unaware of exposure status? |
|  | 0 = No or not reported |
|  | 0.5 = In part |
|  | 1 = Yes |
| E. | Statistical methods |
| 14 | Was the analysis clear and did it use intention-to-treat where applicable? |
|  | 0 = No or not reported |
|  | 0.5 = In part |
|  | 1 = Yes |
| Total score: Q_1_ = sum of above scores / 14 | |
