# Supplementary material for: Effect of methotrexate use on the development of type 2 diabetes in rheumatoid arthritis patients: A systematic review and meta-analysis
Source: PLoS One. 2020 Jul 6;15(7):e0235637. doi: 10.1371/journal.pone.0235637 (PMC7337336; doi:10.1371/journal.pone.0235637)
Supplement: S5 Materials — Quality assessment scores for studies included in the meta-analysis. (PDF) [file pone.0235637.s006.pdf]

B·li
2011

Checklist for assessing quality of included studies in the meta-analysis

A. Design-specific control of bias

1 Was a method of randomisation performed?

0 = No or not reported

1 = yes but allocation not concealed

2 = Yes and allocation concealed

2 Was the data prospectively collected

0 = no - case-control or cross-sectional design

0.5 = no - cohort design retro

1 = yes

B. Selection bias

3 Was the outcome of interest already present at the start of the study?

0 = Yes

1 = No

4 Were protocol deviations, losses to follow-up, and drop-out rates acceptable (<20%)?

0 = No or not reported

0.5 = In part

1 = Yes

5 Were the controls or non-exposed cohort drawn from the same population and in the same way as the exposed cohort?

0 = No or no description

1 = Drawn from a different source

2 = Yes

6 Were the eligibility criteria clearly specified and uniformly applied to comparison groups

0 = No or no description

0.5 = In part

1 = Yes

7 Was selection of a comparison group appropriate?

0 = No or no description

0.5 = In part

1 = Yes

C. Confounding

8 Was any attempt made to balance allocation between groups (excludes randomisation)?

0 = No

0.5 = in part

page 2

1 = yes

9 Were the important prognostic indicators (age, sex, hypertension, BMI, diabetic status, dyslipidaemia, smoking, family history of CVD, physical inactivity, duration of RA, and using medications such as folic acid, corticosteroids, anti-rheumatic medications) of the group/cohorts comparable at baseline and were reported? Were they similar?

0 = not reported or 0,1,2

0.5 = 3, 4

1 = 5 or more

D. Information bias

10 Was there a clear ascertainment of exposure (cohort) or for outcomes or for interventions and were they clearly defined and precisely reported?

0 = No description

0.5 = Self report for exposure or description in part

1 = Yes (e.g. secure record for exposure or structured interview in case of an observational study)

11 Was timing of outcome assessment in both groups and duration of follow-up comparable and adequate for outcomes to occur?

0 = No or not reported

0.5 = In part

1 = Yes

12 Were there variations from study protocol that could have affected study measurements?

0 = No or not reported

0.5 = In part

1 = Yes

13 Were the outcome assessor, care provider, and patients unaware of exposure status?

0 = No or not reported

0.5 = In part

1 = Yes

E. Statistical methods

14 Was the analysis clear and did it use intention-to-treat where applicable?

0 = No or not reported

0.5 = In part

1 = Yes

10

Total score: Q_1 = sum of above scores / 14

Best

2018

Checklist for assessing quality of included studies in the meta-analysis

A. Design-specific control of bias

1 Was a method of randomisation performed?

0 = No or not reported

1 = yes but allocation not concealed

2 = Yes and allocation concealed

2 Was the data prospectively collected

0 = no – case-control or cross-sectional design

0.5= no – cohort design

1=yes

B. Selection bias

3 Was the outcome of interest already present at the start of the study?

0 = Yes

1 = No

4 Were protocol deviations, losses to follow-up, and drop-out rates acceptable (<20%)?

0 = No or not reported

0.5 = In part

1 = Yes

5 Were the controls or non-exposed cohort drawn from the same population and in the same way as the exposed cohort?

0 = No or no description

1 = Drawn from a different source

2 = Yes

6 Were the eligibility criteria clearly specified and uniformly applied to comparison groups

0 = No or no description

0.5 = In part

1 = Yes

7 Was selection of a comparison group appropriate?

0 = No or no description

0.5 = In part

1 = Yes

C. Confounding

8 Was any attempt made to balance allocation between groups (excludes randomisation)?

0 = No

0.5 = in part

page 3

1 = yes

9 Were the important prognostic indicators (age, sex, hypertension, BMI, diabetic status, dyslipidaemia, smoking, family history of CVD, physical inactivity, duration of RA, and using medications such as folic acid, corticosteroids, anti-rheumatic medications) of the group/cohorts comparable at baseline and were reported? Were they similar?

0 = not reported or 0,1,2

0.5 = 3, 4

1 = 5 or more

D. Information bias

10 Was there a clear ascertainment of exposure (cohort) or for outcomes or for interventions and were they clearly defined and precisely reported?

0 = No description

0.5 = Self report for exposure or description in part

no Bl. test values.

for the diagnosis.

or medication use.

1 = Yes (e.g. secure record for exposure or structured interview in case of an observational study)

11 Was timing of outcome assessment in both groups and duration of follow-up comparable and adequate for outcomes to occur?

0 = No or not reported

0.5 = In part

1 = Yes

12 Were there variations from study protocol that could have affected study measurements?

0 = No or not reported

0.5 = In part

1 = Yes

13 Were the outcome assessor, care provider, and patients unaware of exposure status?

0 = No or not reported

0.5 = In part

1 = Yes

E. Statistical methods

14 Was the analysis clear and did it use intention-to-treat where applicable?

0 = No or not reported

0.5 = In part

1 = Yes

Total score: Q_1 = sum of above scores / 14 10

---

chen 2017

Checklist for assessing quality of included studies in the meta-analysis

---

A. Design-specific control of bias

1 Was a method of randomisation performed?

0 = No or not reported

1 = yes but allocation not concealed

2 = Yes and allocation concealed

2 Was the data prospectively collected

0 = no – case-control or cross-sectional design

0.5= no – cohort design Retro

1=yes

B. Selection bias

3 Was the outcome of interest already present at the start of the study?

0 = Yes

1 = No

4 Were protocol deviations, losses to follow-up, and drop-out rates acceptable (<20%)?

0 = No or not reported

0.5 = In part

1 = Yes

5 Were the controls or non-exposed cohort drawn from the same population and in the same way as the exposed cohort?

0 = No or no description

1 = Drawn from a different source

2 = Yes

6 Were the eligibility criteria clearly specified and uniformly applied to comparison groups

0 = No or no description

0.5 = In part

1 = Yes

7 Was selection of a comparison group appropriate?

0 = No or no description

0.5 = In part

1 = Yes

C. Confounding

8 Was any attempt made to balance allocation between groups (excludes randomisation)?

0 = No

0.5 = in part

1 = yes

9 Were the important prognostic indicators (age, sex, hypertension, BMI, diabetic status, dyslipidaemia, smoking, family history of CVD, physical inactivity, duration of RA, and using medications such as folic acid, corticosteroids, anti-rheumatic medications) of the group/cohorts comparable at baseline and were reported? Were they similar?

0 = not reported or 0,1,2

0.5 = 3, 4

1 = 5 or more

D. Information bias

10 Was there a clear ascertainment of exposure (cohort) or for outcomes or for interventions and were they clearly defined and precisely reported?

0 = No description

0.5 = Self report for exposure or description in part

1 = Yes (e.g. secure record for exposure or structured interview in case of an observational study)

11 Was timing of outcome assessment in both groups and duration of follow-up comparable and adequate for outcomes to occur?

0 = No or not reported

0.5 = In part

1 = Yes

12 Were there variations from study protocol that could have affected study measurements?

0 = No or not reported

0.5 = In part

1 = Yes

13 Were the outcome assessor, care provider, and patients unaware of exposure status?

0 = No or not reported

0.5 = In part

1 = Yes

E. Statistical methods

14 Was the analysis clear and did it use intention-to-treat where applicable?

0 = No or not reported

0.5 = In part

1 = Yes

9.5

Total score: Q_1 = sum of above scores / 14

Ruscitti 2019

Checklist for assessing quality of included studies in the meta-analysis

A. Design-specific control of bias

1 Was a method of randomisation performed?

0 = No or not reported

1 = yes but allocation not concealed

2 = Yes and allocation concealed

2 Was the data prospectively collected

0 = no – case-control or cross-sectional design

0.5= no – cohort design

1=yes

B. Selection bias

3 Was the outcome of interest already present at the start of the study?

0 = Yes

1 = No

4 Were protocol deviations, losses to follow-up, and drop-out rates acceptable (<20%)?

0 = No or not reported

0.5 = In part

1 = Yes

5 Were the controls or non-exposed cohort drawn from the same population and in the same way as the exposed cohort?

0 = No or no description

1 = Drawn from a different source

2 = Yes

6 Were the eligibility criteria clearly specified and uniformly applied to comparison groups

0 = No or no description

0.5 = In part

1 = Yes

7 Was selection of a comparison group appropriate?

0 = No or no description

0.5 = In part

1 = Yes

C. Confounding

8 Was any attempt made to balance allocation between groups (excludes randomisation)?

0 = No

0.5 = in part

1 = yes

9 Were the important prognostic indicators (age, sex, hypertension, BMI, diabetic status, dyslipidaemia, smoking, family history of CVD, physical inactivity, duration of RA, and using medications such as folic acid, corticosteroids, anti-rheumatic medications) of the group/cohorts comparable at baseline and were reported? Were they similar?

0 = not reported or 0,1,2

0.5 = 3, 4

1 = 5 or more

D. Information bias

10 Was there a clear ascertainment of exposure (cohort) or for outcomes or for interventions and were they clearly defined and precisely reported?

0 = No description

0.5 = Self report for exposure or description in part

1 = Yes (e.g. secure record for exposure or structured interview in case of an observational study)

11 Was timing of outcome assessment in both groups and duration of follow-up comparable and adequate for outcomes to occur?

0 = No or not reported

0.5 = In part

1 = Yes

12 Were there variations from study protocol that could have affected study measurements?

0 = No or not reported

0.5 = In part

1 = Yes

13 Were the outcome assessor, care provider, and patients unaware of exposure status?

0 = No or not reported

0.5 = In part

1 = Yes

E. Statistical methods

14 Was the analysis clear and did it use intention-to-treat where applicable?

0 = No or not reported

0.5 = In part

1 = Yes

Total score: Q_1 = sum of above scores / 14

Gome 2018

Checkli t for assessing quality of included studies in the meta-analysis

A. Design-specific control of bias

1 Was a method of randomisation performed?

0 = No or not reported

1 = yes but allocation not concealed

2 = Yes and allocation concealed

2 Was the data prospectively collected

0 = no – case-control or cross-sectional design

0.5= no – cohort design

1=yes

B. Selection bias

3 Was the outcome of interest already present at the start of the study?

0 = Yes

1 = No

4 Were protocol deviations, losses to follow-up, and drop-out rates acceptable (<20%)?

0 = No or not reported

0.5 = In part

1 = Yes

5 Were the controls or non-exposed cohort drawn from the same population and in the same way as the exposed cohort?

0 = No or no description

1 = Drawn from a different source

2 = Yes

6 Were the eligibility criteria clearly specified and uniformly applied to comparison groups

0 = No or no description

0.5 = In part

1 = Yes

7 Was selection of a comparison group appropriate?

0 = No or no description

0.5 = In part

1 = Yes

C. Confounding

8 Was any attempt made to balance allocation between groups (excludes randomisation)?

0 = No

0.5 = in part

1 = yes

9 Were the important prognostic indicators (age, sex, hypertension, BMI, diabetic status, dyslipidaemia, smoking, family history of CVD, physical inactivity, duration of RA, and using medications such as folic acid, corticosteroids, anti-rheumatic medications) of the group/cohorts comparable at baseline and were reported? Were they similar?

0 = not reported or 0,1,2

0.5 = 3, 4

1 = 5 or more

D. Information bias

10 Was there a clear ascertainment of exposure (cohort) or for outcomes or for interventions and were they clearly defined and precisely reported?

0 = No description

0.5 = Self report for exposure or description in part

1 = Yes (e.g. secure record for exposure or structured interview in case of an observational study)

11 Was timing of outcome assessment in both groups and duration of follow-up comparable and adequate for outcomes to occur?

0 = No or not reported

0.5 = In part

1 = Yes

12 Were there variations from study protocol that could have affected study measurements?

0 = No or not reported

0.5 = In part

1 = Yes

13 Were the outcome assessor, care provider, and patients unaware of exposure status?

0 = No or not reported

0.5 = In part

1 = Yes

E. Statistical methods

14 Was the analysis clear and did it use intention-to-treat where applicable?

0 = No or not reported

0.5 = In part

1 = Yes

Total score: Q_1 = sum of above scores / 14

Ozen 2017

Checklist for assessing quality of included studies in the meta-analysis

A. Design-specific control of bias

1 Was a method of randomisation performed?

0 = No or not reported

1 = yes but allocation not concealed

2 = Yes and allocation concealed

2 Was the data prospectively collected

0 = no – case-control or cross-sectional design

0.5= no – cohort design

1 = yes

B. Selection bias

3 Was the outcome of interest already present at the start of the study?

0 = Yes

1 = No

4 Were protocol deviations, losses to follow-up, and drop-out rates acceptable (<20%)?

0 = No or not reported

0.5 = In part

1 = Yes

5 Were the controls or non-exposed cohort drawn from the same population and in the same way as the exposed cohort?

0 = No or no description

1 = Drawn from a different source

2 = Yes

6 Were the eligibility criteria clearly specified and uniformly applied to comparison groups

0 = No or no description

0.5 = In part

1 = Yes

7 Was selection of a comparison group appropriate?

0 = No or no description

0.5 = In part

1 = Yes

C. Confounding

8 Was any attempt made to balance allocation between groups (excludes randomisation)?

0 = No

0.5 = in part

1 = yes

9 Were the important prognostic indicators (age, sex, hypertension, BMI, diabetic status, dyslipidaemia, smoking, family history of CVD, physical inactivity, duration of RA, and using medications such as folic acid, corticosteroids, anti-rheumatic medications) of the group/cohorts comparable at baseline and were reported? Were they similar?

0 = not reported or 0,1,2

0.5 = 3, 4

1 = 5 or more

D. Information bias

10 Was there a clear ascertainment of exposure (cohort) or for outcomes or for interventions and were they clearly defined and precisely reported?

0 = No description

0.5 = Self report for exposure or description in part

1 = Yes (e.g. secure record for exposure or structured interview in case of an observational study)

11 Was timing of outcome assessment in both groups and duration of follow-up comparable and adequate for outcomes to occur?

0 = No or not reported

0.5 = In part

1 = Yes

12 Were there variations from study protocol that could have affected study measurements?

0 = No or not reported

0.5 = In part

1 = Yes

13 Were the outcome assessor, care provider, and patients unaware of exposure status?

0 = No or not reported

0.5 = In part

1 = Yes

E. Statistical methods

14 Was the analysis clear and did it use intention-to-treat where applicable?

0 = No or not reported

0.5 = In part

1 = Yes

Total score: Q_1 = sum of above scores / 14

Antohe 2012

Checklist for assessing quality of included studies in the meta-analysis

---

A. Design-specific control of bias

1 Was a method of randomisation performed?

0 = No or not reported

1 = yes but allocation not concealed

2 = Yes and allocation concealed

2 Was the data prospectively collected

0 = no – case-control or cross-sectional design

0.5= no – cohort design

1=yes

B. Selection bias

3 Was the outcome of interest already present at the start of the study?

0 = Yes

1 = No

4 Were protocol deviations, losses to follow-up, and drop-out rates acceptable (<20%)?

0 = No or not reported

0.5 = In part

1 = Yes

5 Were the controls or non-exposed cohort drawn from the same population and in the same way as the exposed cohort?

0 = No or no description

1 = Drawn from a different source

2 = Yes

6 Were the eligibility criteria clearly specified and uniformly applied to comparison groups

0 = No or no description

0.5 = In part

1 = Yes

7 Was selection of a comparison group appropriate?

0 = No or no description

0.5 = In part

1 = Yes

C. Confounding

8 Was any attempt made to balance allocation between groups (excludes randomisation)?

0 = No

0.5 = in part

1 = yes

some how page (5) limitation

page 3 (To avoid excluding pt ē unavailable cohort data test).

9 Were the important prognostic indicators (age, sex, hypertension, BMI, diabetic status, dyslipidaemia, smoking, family history of CVD, physical inactivity, duration of RA, and using medications such as folic acid, corticosteroids, anti-rheumatic medications) of the group/cohorts comparable at baseline and were reported? Were they similar?

0 = not reported or 0,1,2

0.5 = 3, 4

1 = 5 or more

D. Information bias

10 Was there a clear ascertainment of exposure (cohort) or for outcomes or for interventions and were they clearly defined and precisely reported?

0 = No description

0.5 = Self report for exposure or description in part

1 = Yes (e.g. secure record for exposure or structured interview in case of an observational study)

11 Was timing of outcome assessment in both groups and duration of follow-up comparable and adequate for outcomes to occur?

0 = No or not reported

0.5 = In part

1 = Yes

12 Were there variations from study protocol that could have affected study measurements?

0 = No or not reported

0.5 = In part

1 = Yes

13 Were the outcome assessor, care provider, and patients unaware of exposure status?

0 = No or not reported

0.5 = In part

1 = Yes

E. Statistical methods

14 Was the analysis clear and did it use intention-to-treat where applicable?

0 = No or not reported

0.5 = In part

1 = Yes

9

Total score: Q_1 = sum of above scores / 14

Amaya. 2013

Checklist for assessing quality of included studies in the meta-analysis

A. Design-specific control of bias

1 Was a method of randomisation performed?

0 = No or not reported

1 = yes but allocation not concealed

2 = Yes and allocation concealed

2 Was the data prospectively collected

0 = no – case-control or cross-sectional design

0.5= no – cohort design

1=yes

B. Selection bias

3 Was the outcome of interest already present at the start of the study?

0 = Yes

1 = No

4 Were protocol deviations, losses to follow-up, and drop-out rates acceptable (<20%)?

0 = No or not reported

0.5 = In part

1 = Yes

5 Were the controls or non-exposed cohort drawn from the same population and in the same way as the exposed cohort?

0 = No or no description

1 = Drawn from a different source

2 = Yes

6 Were the eligibility criteria clearly specified and uniformly applied to comparison groups

0 = No or no description

0.5 = In part

1 = Yes

7 Was selection of a comparison group appropriate?

0 = No or no description

0.5 = In part

1 = Yes

C. Confounding

8 Was any attempt made to balance allocation between groups (excludes randomisation)?

0 = No

0.5 = in part

1 = yes

9 Were the important prognostic indicators (age, sex, hypertension, BMI, diabetic status, dyslipidaemia, smoking, family history of CVD, physical inactivity, duration of RA, and using medications such as folic acid, corticosteroids, anti-rheumatic medications) of the group/cohorts comparable at baseline and were reported? Were they similar?

0 = not reported or 0,1,2

0.5 = 3, 4

1 = 5 or more

D. Information bias

10 Was there a clear ascertainment of exposure (cohort) or for outcomes or for interventions and were they clearly defined and precisely reported?

0 = No description

0.5 = Self report for exposure or description in part

1 = Yes (e.g. secure record for exposure or structured interview in case of an observational study)

11 Was timing of outcome assessment in both groups and duration of follow-up comparable and adequate for outcomes to occur?

0 = No or not reported

0.5 = In part

1 = Yes

12 Were there variations from study protocol that could have affected study measurements?

0 = No or not reported

0.5 = In part

1 = Yes

13 Were the outcome assessor, care provider, and patients unaware of exposure status?

0 = No or not reported

0.5 = In part

1 = Yes

E. Statistical methods

14 Was the analysis clear and did it use intention-to-treat where applicable?

0 = No or not reported

0.5 = In part

1 = Yes

Total score: Q_1 = sum of above scores 9 / 14

Ag Ca 2019

MTX → DMARDs.

T2D.

Checklist for assessing quality of included studies in the meta-analysis

A. Design-specific control of bias

1 Was a method of randomisation performed?

0 = No or not reported

1 = yes but allocation not concealed

2 = Yes and allocation concealed

2 Was the data prospectively collected

0 = no – case-control or cross-sectional design

0.5 = no – cohort design

1 = yes

B. Selection bias

3 Was the outcome of interest already present at the start of the study?

0 = Yes

PCS

1 = No

4 Were protocol deviations, losses to follow-up, and drop-out rates acceptable (<20%)?

0 = No or not reported

0.5 = In part

it is 24% in comparison group and 2.5% in exp. group.

1 = Yes

5 Were the controls or non-exposed cohort drawn from the same population and in the same way as the exposed cohort?

NO

0 = No or no description

1 = Drawn from a different source

2 = Yes

6 Were the eligibility criteria clearly specified and uniformly applied to comparison groups

0 = No or no description

0.5 = In part

1 = Yes

7 Was selection of a comparison group appropriate?

0 = No or no description

0.5 = In part

1 = Yes

C. Confounding

8 Was any attempt made to balance allocation between groups (excludes randomisation)?

0 = No

0.5 = in part

1 = yes

9 Were the important prognostic indicators (age, sex, hypertension, BMI, diabetic status, dyslipidaemia, smoking, family history of CVD, physical inactivity, duration of RA, and using medications such as folic acid, corticosteroids, anti-rheumatic medications) of the group/cohorts comparable at baseline and were reported? Were they similar? page 7.

0 = not reported or 0,1,2

0.5 = 3, 4

1 = 5 or more

D. Information bias

10 Was there a clear ascertainment of exposure (cohort) or for outcomes or for interventions and were they clearly defined and precisely reported?

0 = No description

0.5 = Self report for exposure or description in part

1 = Yes (e.g. secure record for exposure or structured interview in case of an observational study)

11 Was timing of outcome assessment in both groups and duration of follow-up comparable and adequate for outcomes to occur? 15 y. CVD

0 = No or not reported

0.5 = In part

1 = Yes

12 Were there variations from study protocol that could have affected study measurements?

0 = No or not reported 15

0.5 = In part

1 = Yes

13 Were the outcome assessor, care provider, and patients unaware of exposure status?

0 = No or not reported

0.5 = In part

1 = Yes

E. Statistical methods

14 Was the analysis clear and did it use intention-to-treat where applicable?

0 = No or not reported

0.5 = In part

1 = Yes

10.5

Total score: Q_1 = sum of above scores / 14

Mangoni&Baghdadi et al. 201[illegible]

Checklist for assessing quality of included studies in the meta-analysis

---

A. Design-specific control of bias

1 Was a method of randomisation performed?

0 = No or not reported

1 = yes but allocation not concealed

2 = Yes and allocation concealed

2 Was the data prospectively collected

0 = no – case-control or cross-sectional design

0.5= no – cohort design

1 = yes

B. Selection bias

3 Was the outcome of interest already present at the start of the study?

0 = Yes

1 = No

4 Were protocol deviations, losses to follow-up, and drop-out rates acceptable (<20%)?

0 = No or not reported

0.5 = In part

1 = Yes

5 Were the controls or non-exposed cohort drawn from the same population and in the same way as the exposed cohort?

0 = No or no description

1 = Drawn from a different source

2 = Yes

6 Were the eligibility criteria clearly specified and uniformly applied to comparison groups

0 = No or no description

0.5 = In part

1 = Yes

7 Was selection of a comparison group appropriate?

0 = No or no description

0.5 = In part

1 = Yes

C. Confounding

8 Was any attempt made to balance allocation between groups (excludes randomisation)?

0 = No

0.5 = in part

age. medication

1 = yes

9 Were the important prognostic indicators (age, sex, hypertension, BMI, diabetic status, dyslipidaemia, smoking, family history of CVD, physical inactivity, duration of RA, and using medications such as folic acid, corticosteroids, anti-rheumatic medications) of the group/cohorts comparable at baseline and were reported? Were they similar?

0 = not reported or 0,1,2

0.5 = 3, 4

1 = 5 or more

D. Information bias

10 Was there a clear ascertainment of exposure (cohort) or for outcomes or for interventions and were they clearly defined and precisely reported?

0 = No description

0.5 = Self report for exposure or description in part

1 = Yes (e.g. secure record for exposure or structured interview in case of an observational study)

11 Was timing of outcome assessment in both groups and duration of follow-up comparable and adequate for outcomes to occur?

0 = No or not reported

0.5 = In part

1 = Yes

12 Were there variations from study protocol that could have affected study measurements?

0 = No or not reported

0.5 = In part

1 = Yes

13 Were the outcome assessor, care provider, and patients unaware of exposure status?

0 = No or not reported

0.5 = In part

1 = Yes

E. Statistical methods

14 Was the analysis clear and did it use intention-to-treat where applicable?

0 = No or not reported

0.5 = In part

1 = Yes

Total score: Q_1 = sum of above scores / 14

Wasko 2007

Table S1. Checklist for assessing quality of included studies in the meta-analysis

A. Design-specific control of bias

1 Was a method of randomisation performed?

0 = No or not reported

1 = yes but allocation not concealed

2 = Yes and allocation concealed

2 Was the data prospectively collected

0 = no – case-control or cross-sectional design

0.5 = no – cohort design

1 = yes

B. Selection bias

3 Was the outcome of interest already present at the start of the study?

0 = Yes

1 = No

4 Were protocol deviations, losses to follow-up, and drop-out rates acceptable (<20%)?

0 = No or not reported

0.5 = In part

1 = Yes

5 Were the controls or non-exposed cohort drawn from the same population and in the same way as the exposed cohort?

0 = No or no description

1 = Drawn from a different source

2 = Yes

6 Were the eligibility criteria clearly specified and uniformly applied to comparison groups

0 = No or no description

0.5 = In part

1 = Yes

7 Was selection of a comparison group appropriate?

0 = No or no description

0.5 = In part

1 = Yes

C. Confounding

8 Was any attempt made to balance allocation between groups (excludes randomisation)?

0 = No

0.5 = in part

1 = yes

9 Were the important prognostic indicators (age, sex, hypertension, BMI, diabetic status, dyslipidaemia, smoking, family history of CVD, physical inactivity, duration of RA, and using medications such as folic acid, corticosteroids, anti-rheumatic medications) of the group/cohorts comparable at baseline and were reported? Were they similar?

0 = not reported or 0,1,2

0.5 = 3, 4

1 = 5 or more

D. Information bias

10 Was there a clear ascertainment of exposure (cohort) or for outcomes or for interventions and were they clearly defined and precisely reported?

0 = No description

0.5 = Self report for exposure or description in part

1 = Yes (e.g. secure record for exposure or structured interview in case of an observational study)

11 Was timing of outcome assessment in both groups and duration of follow-up comparable and adequate for outcomes to occur?

0 = No or not reported

0.5 = In part

1 = Yes

12 Were there variations from study protocol that could have affected study measurements?

0 = No or not reported

0.5 = In part

1 = Yes

13 Were the outcome assessor, care provider, and patients unaware of exposure status?

0 = No or not reported

0.5 = In part

1 = Yes

E. Statistical methods

14 Was the analysis clear and did it use intention-to-treat where applicable?

0 = No or not reported

0.5 = In part

1 = Yes

10

Total score: Q_1 = sum of above scores / 14

Sdomon 2011

J-AP [illegible]

Checklist for assessing quality of included studies in the meta-analysis

A. Design-specific control of bias

1 Was a method of randomisation performed?

0 = No or not reported

1 = yes but allocation not concealed

2 = Yes and allocation concealed

2 Was the data prospectively collected

0 = no – case-control or cross-sectional design

0.5= no – cohort design

1=yes

B. Selection bias

3 Was the outcome of interest already present at the start of the study?

0 = Yes

1 = No

4 Were protocol deviations, losses to follow-up, and drop-out rates acceptable (<20%)?

0 = No or not reported

0.5 = In part

1 = Yes

5 Were the controls or non-exposed cohort drawn from the same population and in the same way as the exposed cohort?

0 = No or no description

1 = Drawn from a different source

2 = Yes

6 Were the eligibility criteria clearly specified and uniformly applied to comparison groups

0 = No or no description

0.5 = In part

1 = Yes

7 Was selection of a comparison group appropriate?

0 = No or no description

0.5 = In part

1 = Yes

C. Confounding

8 Was any attempt made to balance allocation between groups (excludes randomisation)?

0 = No

0.5 = in part

1 = yes

9 Were the important prognostic indicators (age, sex, hypertension, BMI, diabetic status, dyslipidaemia, smoking, family history of CVD, physical inactivity, duration of RA, and using medications such as folic acid, corticosteroids, anti-rheumatic medications) of the group/cohorts comparable at baseline and were reported? Were they similar?

0 = not reported or 0,1,2

0.5 = 3, 4

1 = 5 or more

D. Information bias

10 Was there a clear ascertainment of exposure (cohort) or for outcomes or for interventions and were they clearly defined and precisely reported?

0 = No description

0.5 = Self report for exposure or description in part

1 = Yes (e.g. secure record for exposure or structured interview in case of an observational study)

11 Was timing of outcome assessment in both groups and duration of follow-up comparable and adequate for outcomes to occur?

0 = No or not reported

It was 5.8 months

0.5 = In part

1 = Yes

12 Were there variations from study protocol that could have affected study measurements?

0 = No or not reported

0.5 = In part

1 = Yes

13 Were the outcome assessor, care provider, and patients unaware of exposure status?

0 = No or not reported

0.5 = In part

1 = Yes

E. Statistical methods

14 Was the analysis clear and did it use intention-to-treat where applicable?

0 = No or not reported

0.5 = In part

1 = Yes

10

Total score: Q_1 = sum of above scores / 14
