# Supplementary material for: Effect of methotrexate use on the development of type 2 diabetes in rheumatoid arthritis patients: A systematic review and meta-analysis
Source: PLoS One. 2020 Jul 6;15(7):e0235637. doi: 10.1371/journal.pone.0235637 (PMC7337336; doi:10.1371/journal.pone.0235637)
Supplement: S1 Dataset — (PDF) [file pone.0235637.s007.pdf]

QE Model Risk of T2D
RE Model Risk of T2D
FE Model Risk of T2D

					MTX			None MTX				
spontane	Article No	type of outcome	study yea	study name	N1	Cases	Non-case	N2	Cases	Non-case	Qi	Raw score
1	1	T2D	2017	Chen et al	44635	1137	43498	37547	1338	36209	0.678571	9.5
1	2	T2D	2017	Ozen et al	9686	765	8921	3983	374	3609	0.785714	11
1	3	T2D	2011	Solomon et al	7614	82	7532	3713	55	3658	0.714286	10
1	4	T2D	2007	Wasko et al	3097	171	2926	653	54	599	0.714286	10
1	5	T2D	2012	Antohe et al	592	49	543	121	16	105	0.642857	9
1	6	T2D	2007	Assous et al	201	6	195	36	12	24	0.5	7
1	7	T2D	2009	Radovits et al	112	4	108	110	14	96	0.785714	11
1	8	T2D	2017	Mangoni et al	57	3	54	30	5	25	0.857143	12
1	9	T2D	2019	Agca et al	195	3	192	131	13	181	0.75	10.5
1	10	T2D	2011	Bili et al	482	3	479	312	42	270	0.714286	10
1	11	T2D	2018	Best et al	20873	7	20866	23317	6446	16871	0.714286	10
1	12	T2D	2001	del Rincon et al	150	10	140	86	28	58	0.785714	11
1	13	T2D	2018	Gomes et al	203	12	191	135	49	86	0.535714	7.5
1	14	T2D	2011	Innala et al	361	8	353	81	33	48	0.785714	11
1	15	T2D	2019	Ruscitti et al	716	10	706	95	88	7	0.785714	11
1	16	T2D	2013	Amaya-Amaya et al	702	4	698	35	28	7	0.642857	9
